# Supplementary figures and images for: Targeted chromosomal Escherichia coli:dnaB exterior surface residues regulate DNA helicase behavior to maintain genomic stability and organismal fitness
Source: PLoS Genet. 2021 Nov 12;17(11):e1009886. doi: 10.1371/journal.pgen.1009886 (PMC8612530; doi:10.1371/journal.pgen.1009886)

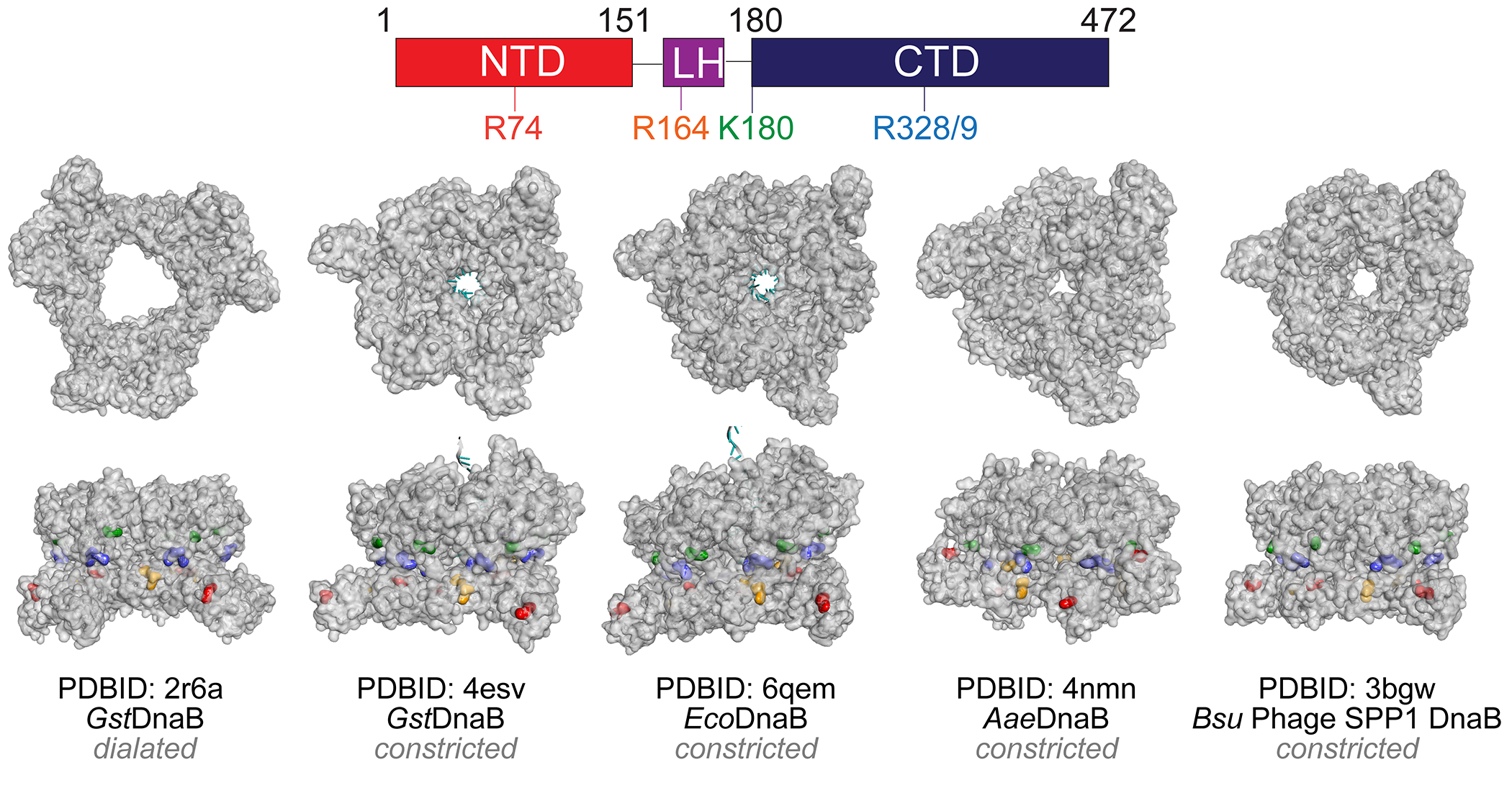

Supplement: S1 Fig — Crystal structure of dilated DnaB (PDB: 2r6a), constricted cracked DnaB (lockwasher, PDB: 6qem), and constricted DnaB (PDB: 3bgw) with mutated residues highlighted: R74A in red, R164A in orange, K180A in green, and R328/9A in blue. The linear protein map (top) shows the location of each mutation relative to functional domains. (TIF) [file pgen.1009886.s004.tif]

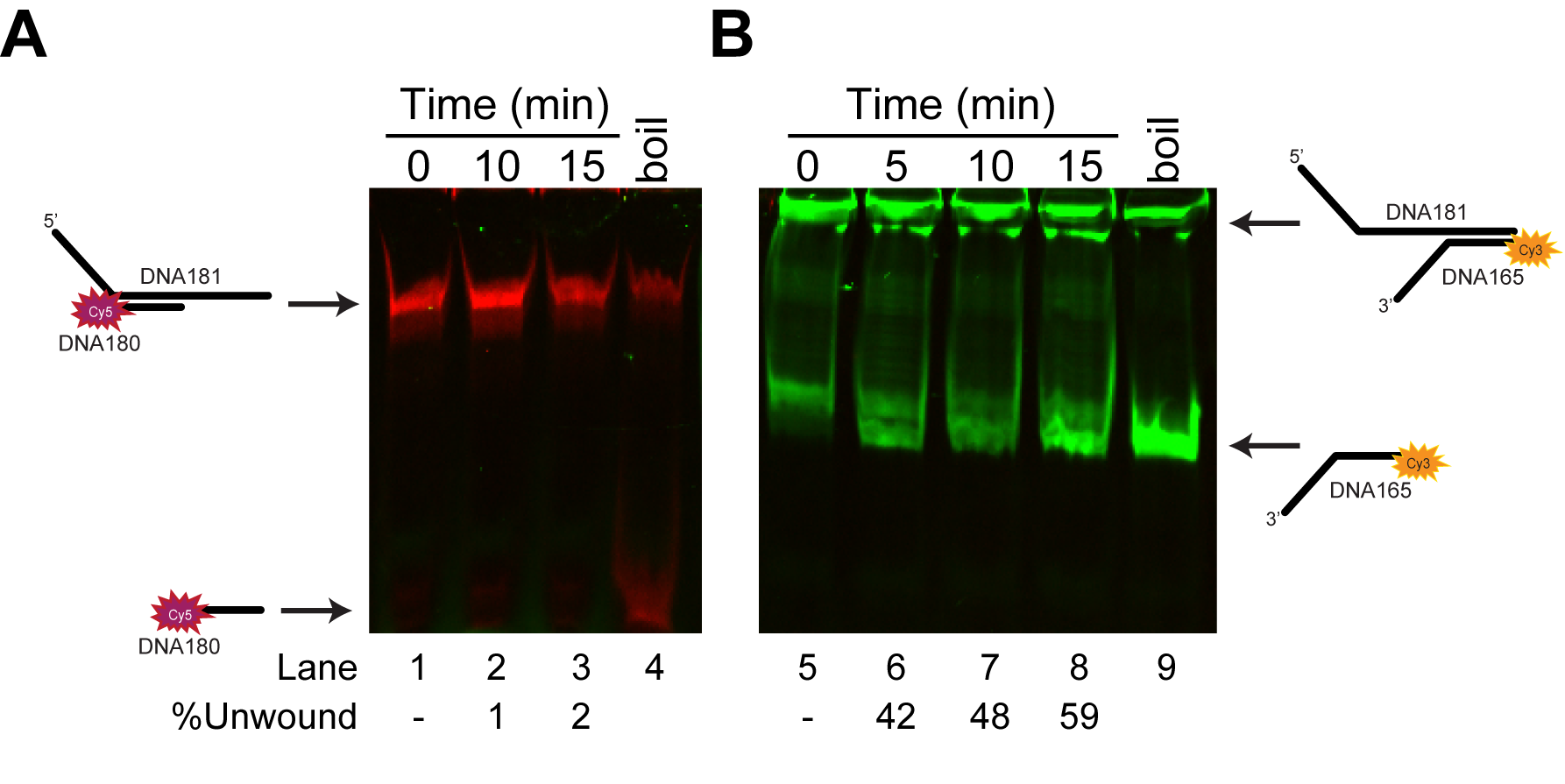

Supplement: S2 Fig — DNA duplex unwinding by WT DnaB (500 nM monomers) to ensure (A) translocation over DNA180 without displacement and (B) unwinding and separation of the DNA180/181 fork. 20 nM of annealed DNA substrate was incubated with 500 nM DnaB (monomers) for 5 minutes at 37°C, initiated with 1 mM ATP and 150 nM respective unlabeled trap strand, and then EDTA quenched with 150 nM trap strand at indicated time points. %Unwound is indicated below each lane. (TIF) [file pgen.1009886.s005.tif]

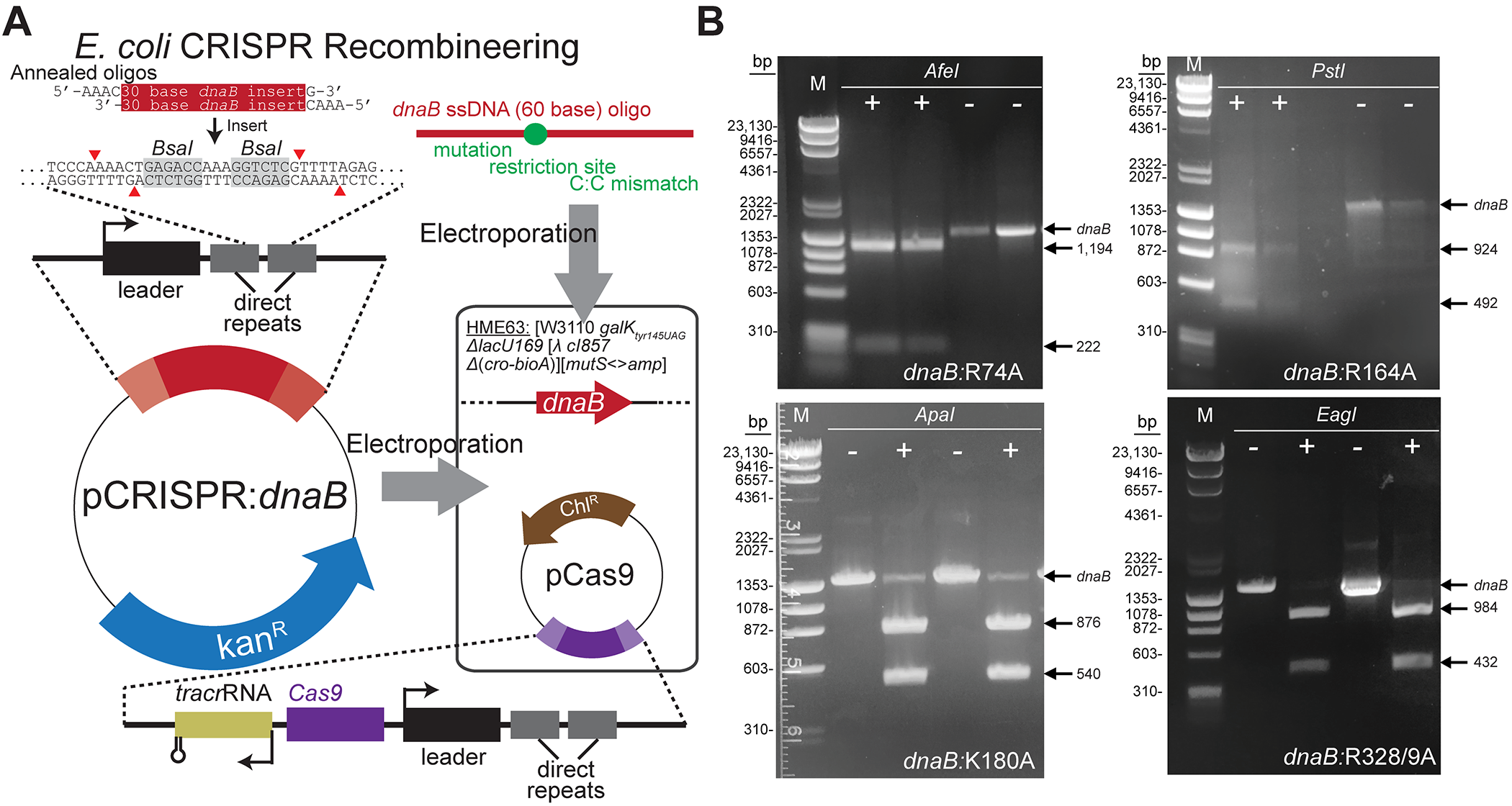

Supplement: S3 Fig — (A) CRISPR-Cas9 recombineering using the dual-plasmid system. A target gRNA for the desired mutation site on the dnaB gene was inserted into the BsaI cloning sites of pCRISPR, before electroporating both the pCRISPR plasmid and the recombination DNA oligonucleotide, engineered to contain the mutation, a novel restriction enzyme site for screening, and a point mutation to disrupt the PAM (5’-NGG) sequence. (B) Restriction digest gels for each of the engineered dnaB mutants showing successful digest at the novel restriction site and confirming dnaB gene mutation for several colonies. The frequencies of positively edited dnaB were 91% for R74A, 83% for R164A, 42% for K180A, and 69% for R328/9A. (TIF) [file pgen.1009886.s006.tif]

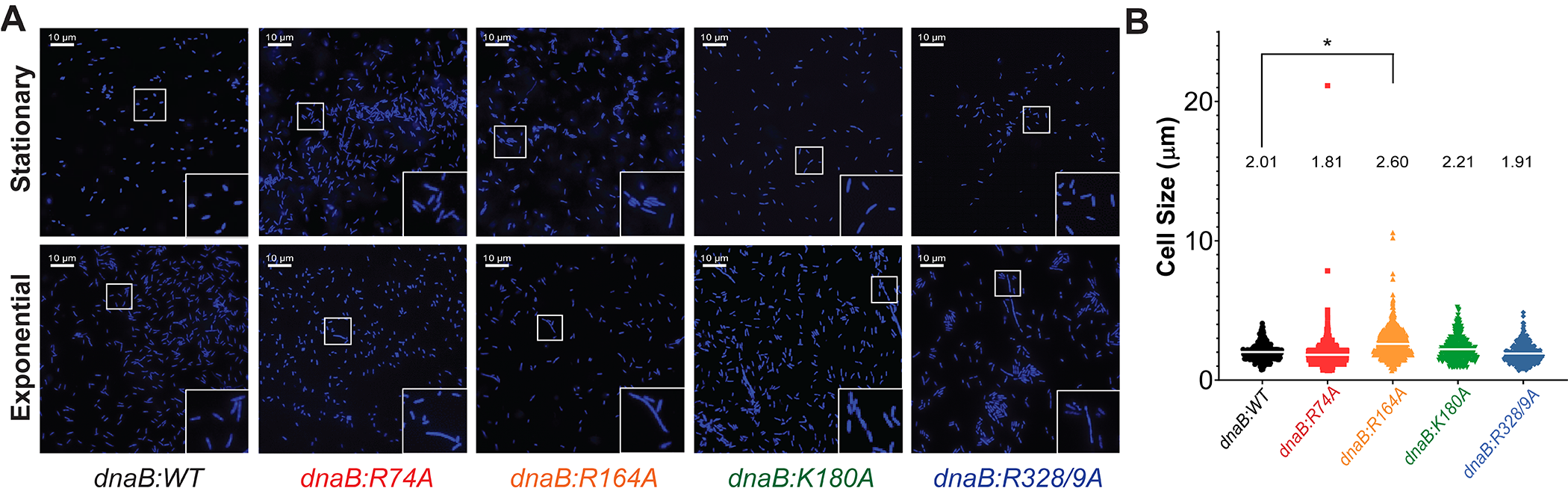

Supplement: S4 Fig — (A) Exponentially growing cells or overnight stationary phase cultures were imaged by microscopy, and (B) stational phase cell lengths were measured by blinded visual quantification. Average cell length is represented by the black bar in the middle of the data set. Average length in order from left to right: 2.0 ± 0.5 μm, 1.9 ± 0.6 μm, 2.6 ± 0.9 μm, 2.2 ± 0.8 μm, and 1.9 ± 0.7 μm. n ≥ 400 events. Black bars above graph indicate statistically significant differences, where p-values are *< 0.05. (TIF) [file pgen.1009886.s007.tif]

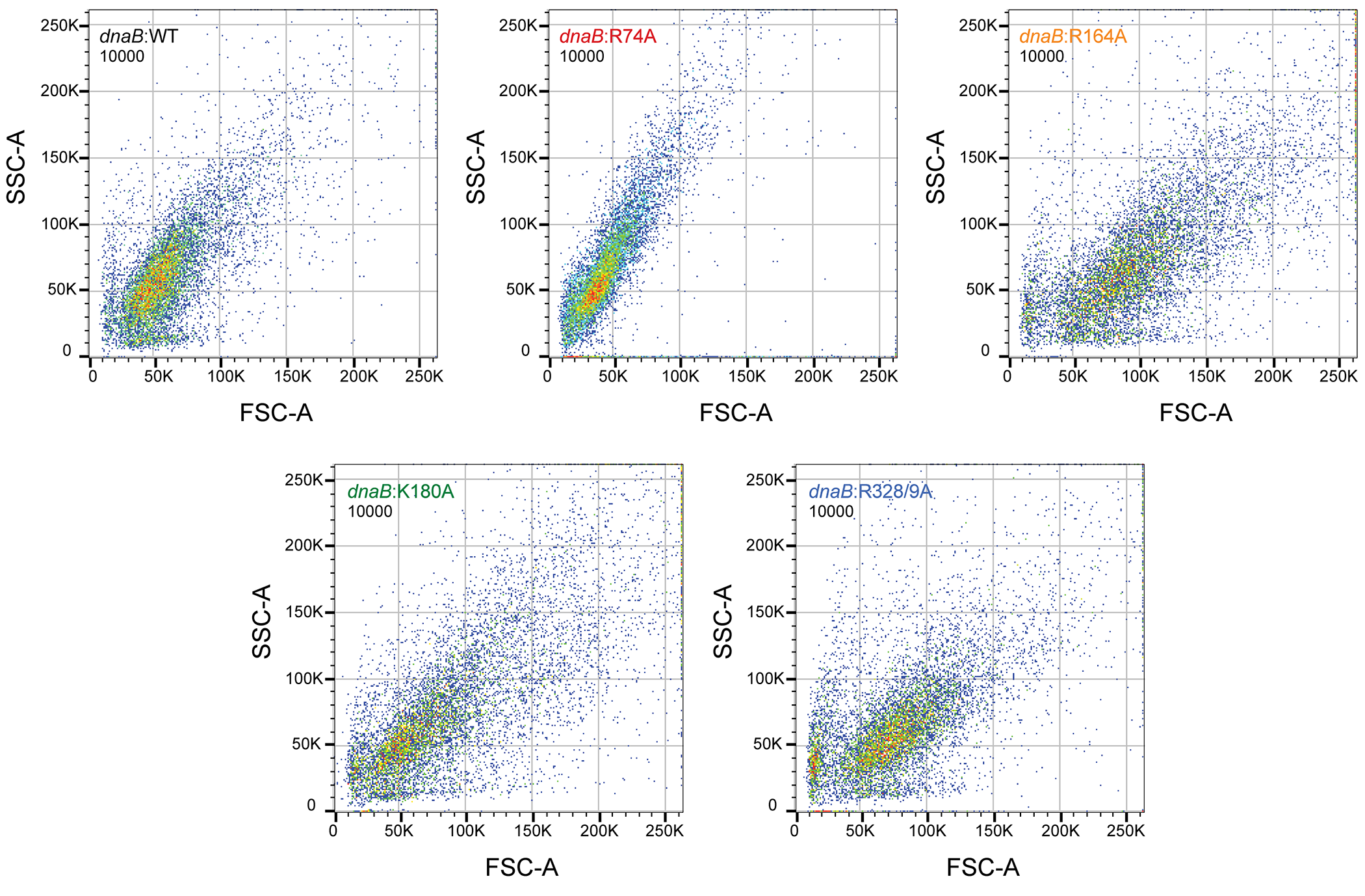

Supplement: S5 Fig — Cell cultures exposed to rifampicin and cephalexin were then analyzed by FACS. The forward (FSC) and side scatter (SSC) gains were set based on the parental strain, and then scatter plot data was collected for all strains, n = 10,000 events. Dense populations of cells are indicated with red, moderate with green, and minor or diffuse population with blue. The parental strain has a single cell population that is primarily green. dnaB:R74A has a single population that is the most concentrated of all the strains (including parental), signified by strong red signal. dnaB:R164A, dnaB:K180A, and dnaB:R328/9A are significantly more diffuse than the parental and dnaB:R74A. (TIF) [file pgen.1009886.s008.tif]

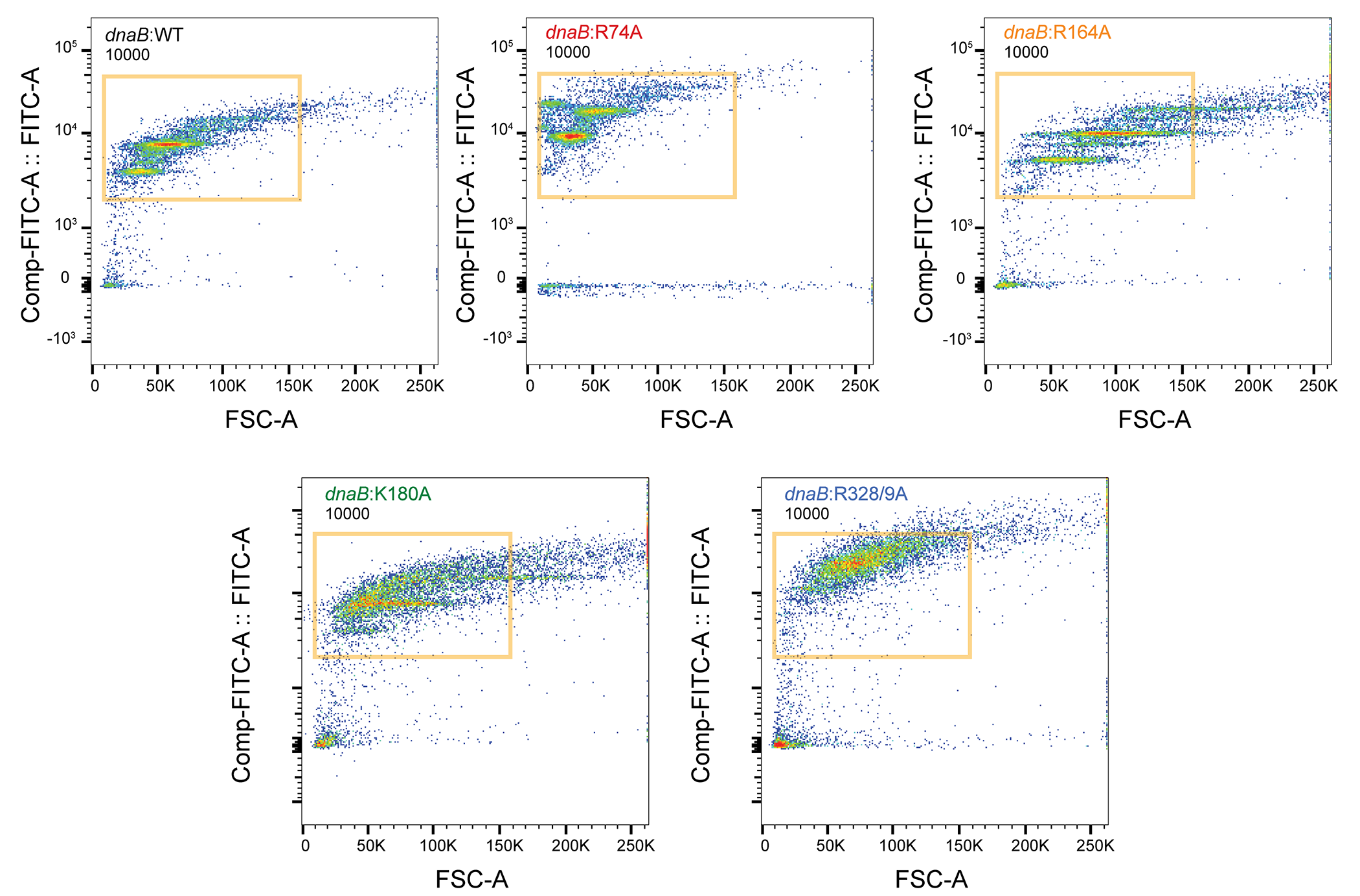

Supplement: S6 Fig — FITC versus FSC (forward scatter) plot of cell cultures exposed to rifampicin and cephalexin analyzed by FACS. FITC indicates the chromatin staining intensity, and FSC indicates cell size. Dense populations of cells are indicated with red, moderate with green, and minor or diffuse population with blue. Fixed yellow box is intended to highlight shift in the location of populations. The parental strain has two major cell populations, with cell size increasing with chromatin. dnaB:R74A is shifted to the left, with small cells containing large amounts of chromatin. dnaB:R164A has three elongated FITC populations, meaning that a single concentration of chromatin is contained within a wide range of cell sizes. The cell populations of dnaB:K180A have lost definition, diffusing into one another. dnaB:R328/9A only has a single large population near the top of the FITC axis, indicating that this strain contains almost exclusively varied cell sizes with large amounts of chromatin. (TIF) [file pgen.1009886.s009.tif]

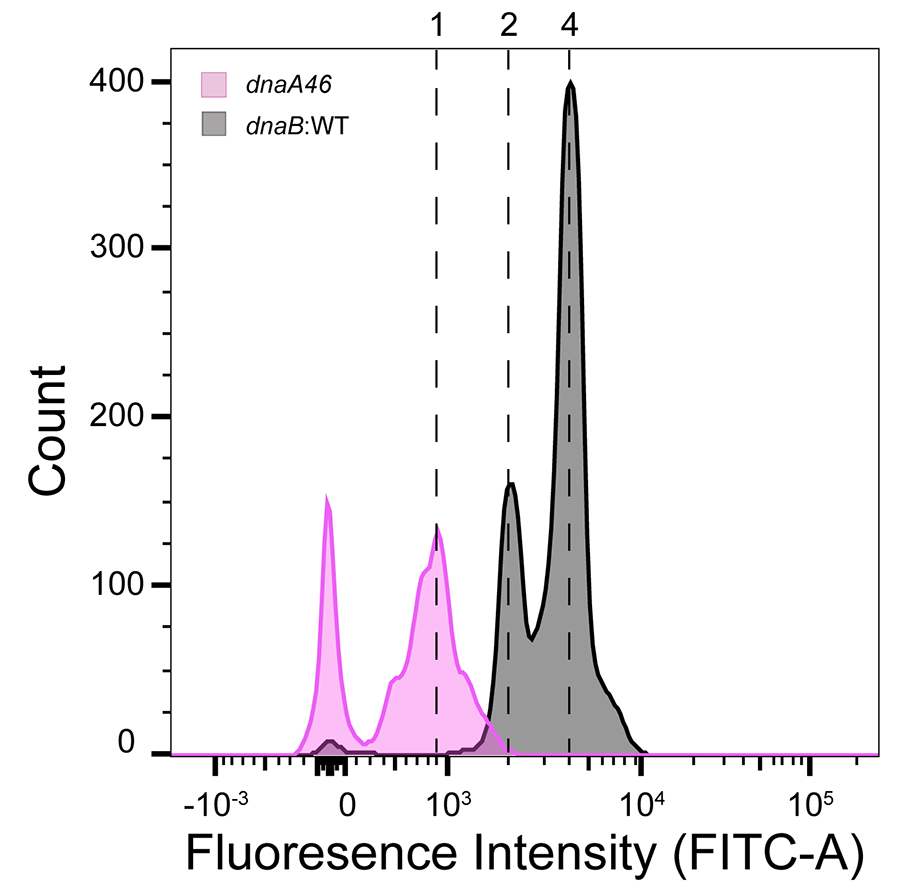

Supplement: S7 Fig — A control experiment measuring chromosome density for the parental strain dnaB:WT and a single-chromosome strain, dnaA46(Ts). Chromosome density was measured by flow cytometry (FACS) in log phase rifampicin ‘run-out’ cultures stained with Sytox Green (n = 10,000 events). dnaA46 was grown at the nonpermissive temperature (42°C) to synchronize the culture before FACS analysis as described in the Materials and Methods. Chromosome integers are indicated at the top of the graph. (TIF) [file pgen.1009886.s010.tif]

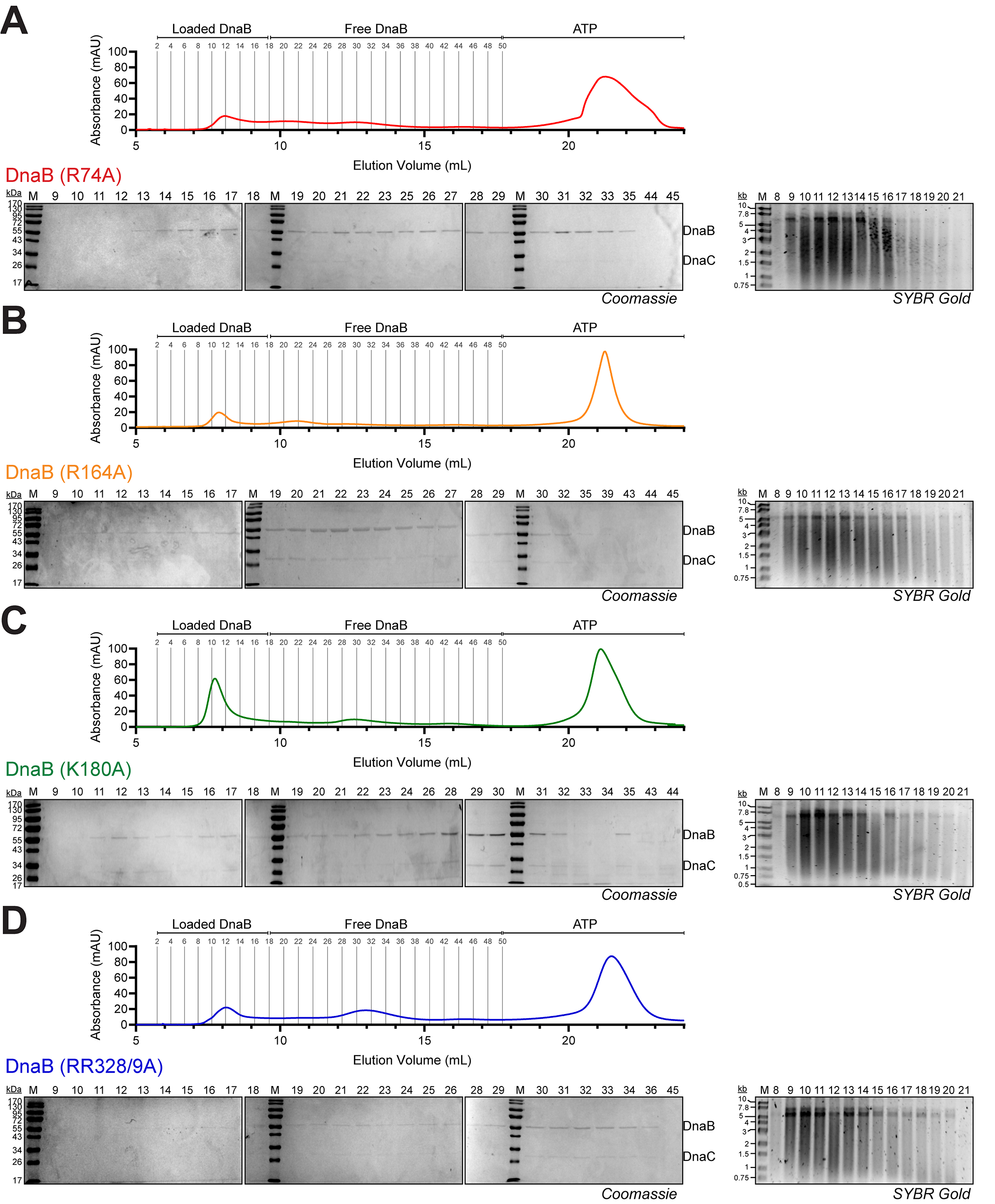

Supplement: S8 Fig — DnaB (A) R74A, (B) R164A, (C) K180A, and (D) R328/9A were preincubated with DnaC, M13, and ATP before injecting onto a preequilibrated S200 10/30 size exclusion column according to the Materials and Methods. Example chromatogram and associated SDS-PAGE (Coomassie) and agarose (SYBR-Gold) gels used to monitor loaded DnaB and Free DnaB areas. (TIF) [file pgen.1009886.s011.tif]

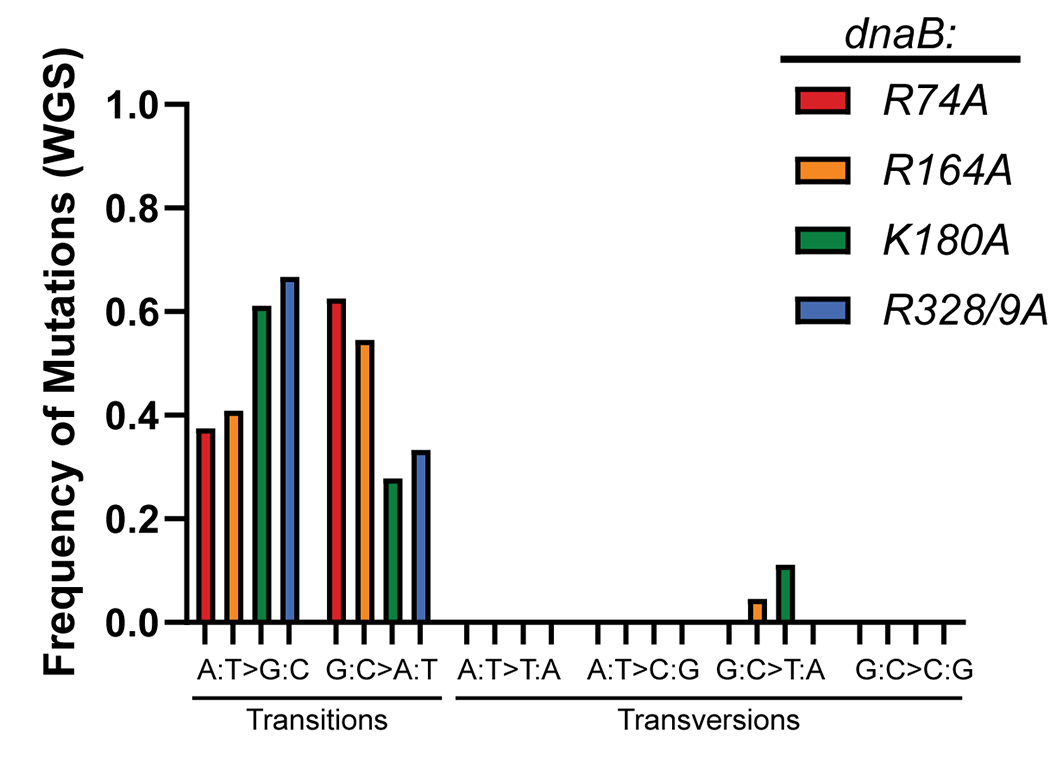

Supplement: S9 Fig — Classification and characterization of transition (Ts) and transversion (Tv) genomic mutations in the dnaB:muts strains. (TIF) [file pgen.1009886.s012.tif]

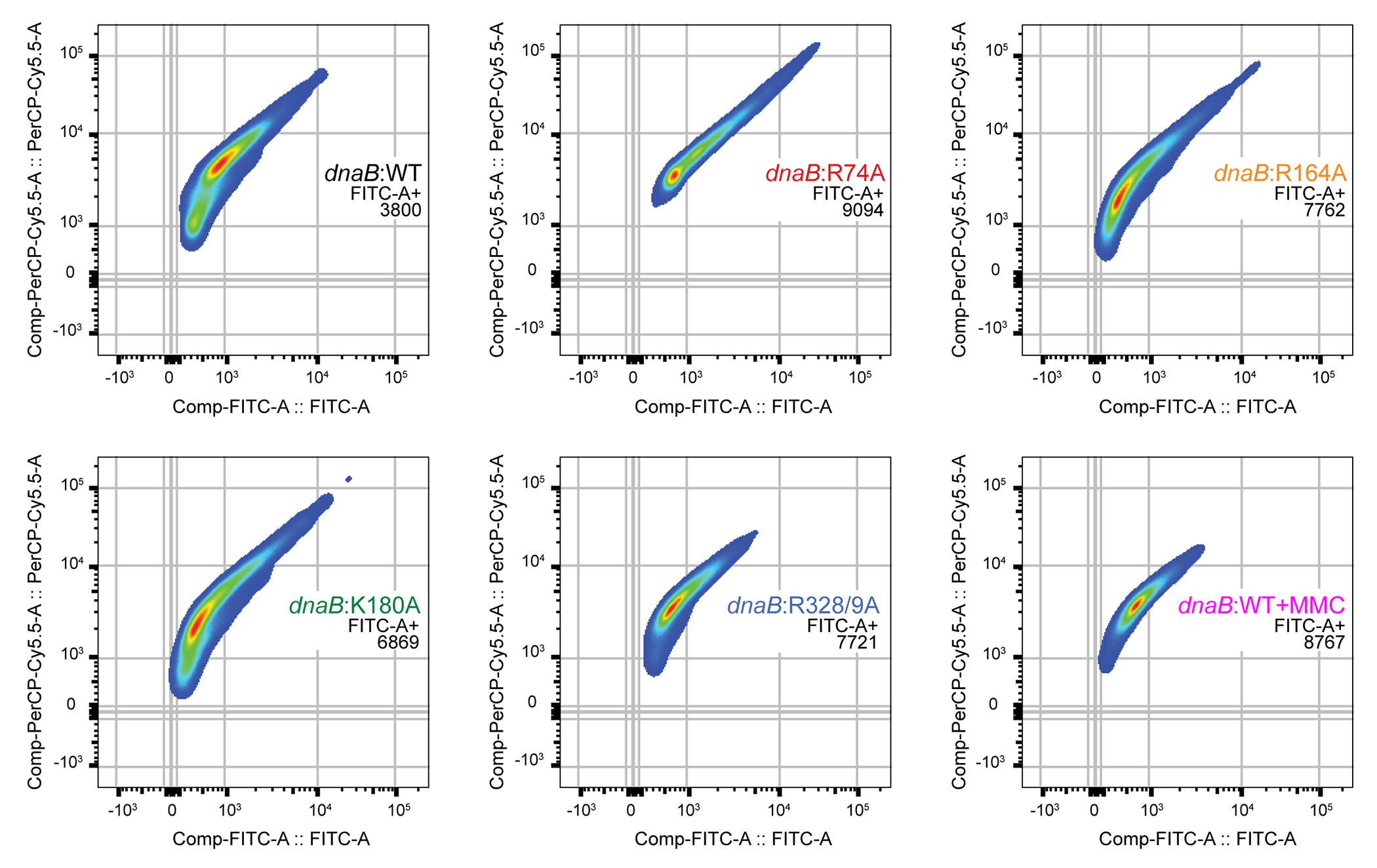

Supplement: S10 Fig — Flow cytometry data of log phase cells plotted to show the relationship between DNA breaks and total amount of DNA. In these smoothed density plots, red represents concentrated cell populations, and dark blue represents highly diffuse cell populations. The grid is present to highlight changes in the size or location of cell populations. Small overall areas indicate concentrated populations that have a uniform and consistent distribution of DNA breaks (as that seen for parent + MMC). Noticeable tailing relative to the parental strain represents populations of cells that have increased DNA and DNA damage (as seen for dnaB:R74A). Y-axis shifts indicate changes in DNA repair or damage sensitivity; while X-axis shifts indicate changes in the amount of chromatin. (TIF) [file pgen.1009886.s013.tif]
