# Supplementary material for: Targeted chromosomal Escherichia coli:dnaB exterior surface residues regulate DNA helicase behavior to maintain genomic stability and organismal fitness
Source: PLoS Genet. 2021 Nov 12;17(11):e1009886. doi: 10.1371/journal.pgen.1009886 (PMC8612530; doi:10.1371/journal.pgen.1009886)
Supplement: Attachment — Submitted filename: Reviewer comments final.pdf [file pgen.1009886.s014.pdf]

Reviewer #1:

Summary: In this manuscript Behrmann et al., address the importance of helicase regulation in replication and genome maintenance. DnaB steric exclusion and wrapping (SEW) mutants result in faster unwinding of DNA in vitro. The authors use four mutants on this interface to ask what the impact of DnaB mis-regulation is on DNA replication and genome stability in vivo. They use an in vitro duplexed fluorescence translocation fork assay to first assess which conformation the SEW mutants adopt and conclude that these are likely to be more locked in the constricted state (in line with the increased fork speed measured in vitro). However, it is apparent that the four mutants behave somewhat distinctly: the authors introduce the mutants in vivo to assess the impact on cellular fitness, replication and genome stability. They find that strains carrying the mutants are compromised in growth and display heterogenous cell size distributions. Indeed, each mutant also impacts chromosome copy number and ori:ter ratio in distinct manners with the R328/9A mutant having the most pronounced effect on replication completion. Counterintuitively, this mutant is significantly compromised in loading on ssDNA. Given the impact of the mutants on cell growth and replication, the authors hypothesize an impact of the mutants on genome integrity. They find that two mutants resulted in increased mutagenesis. On the other hand, SOS response was activated in only R328/9A. However, unlike the SOS activation, all mutants showed likely increase in DSBs. The authors conclude that DnaB regulation via SEW interface is important for genome stability and efficient replication.

Strengths: Behrmann et al., ask an important question about helicase regulation and the coupling of helicase progression with the rest of the replisome in steady state growth conditions. They take a nice approach of testing the impact of well-characterized in vitro mutants on in vivo function. Their study reveals the significance of this interface on helicase regulation and the impact of the same on genome maintenance.

Limitations: While the approaches used by the authors provide support to the idea that helicase regulation is important, the results do not provide a definitive understanding of how mis-regulation may impact DNA replication and / or genome stability. This is in part due to the distinct phenotypes of the mutants. Thus, it remains unclear as to how exactly the regulation of the constriction-dilation states affects DNA replication and why this might in turn impact genome stability. For example, are the effects of the mutants distinct due to differences in the lengths of ssDNA gaps on the chromosome? It is possible that the genome instability phenotype is caused due to RecA mis-loading on long ssDNA tracts in some cases. Similarly, it is unclear as to how and why DSBs are generated each of the mutant contexts. Indeed, answers to some of these open questions may be outside the scope of the present study.

Thank you for recognizing the strengths of this work. We also feel that the apparent limitations seen with the different mutations provides a spectrum of deleterious effects that underscore the role of the helicase in coordinating activities at the replisome to prevent

genome instability. Many of the specific questions raised by all three reviewers are also of great interest to us. We have made a concerted attempt to address most of the questions raised by the reviewers that include new experiments and data that should strengthen this manuscript, further clarify unanswered questions, and more significantly show the importance of these helicase mutations in maintaining cellular fitness and genome stability.

These new experiments include:

1) Whole genome sequencing of the initial strains establishing that the *dnaB:mut* strains are classified as mutator strains that show several random mutations across the genome and the absence of any obvious suppressors.
2) Deletions of *recA* in *dnaB:mut* strains that either show slower growth phenotypes or synthetic lethality (for review only).
3) HU treatment negative control for the TUNEL assay showing minimal staining to validate the assay
4) Further plate reader GFP SOS assays in undamaged conditions and a confirmation western blot that show no significant SOS induction in nontreated conditions.
5) Updated rifampicin assay in YB show natural mutational frequencies
6) Control experiments for the dilated/constricted unwinding assay

Major comments: I have the following specific comments that the authors should address in order to strengthen/ clarify observations in the present study.

1. Authors should combine the dnaB mutants with deletions of recA and separately recO (or recR) to assess for synthetic lethality or if the absence of RecA loading can alleviate the phenotypes observed in the mutant(s).
This was also mentioned by Reviewer 3 and so we felt obligated to do these experiments. We have attempted to make *recA* deletion constructs using FRT:KAN:FRT recombination in our *dnaB:mut* strains. Although we could readily make the *recA* knockout in the WT strain, *recA* knockouts in each of the *dnaB:mut* strains were more difficult. After several trials, we were only able to get single confirmed *ΔrecA* recombinant colonies in *dnaB:R74A*, *dnaB:R164A*, and *dnaB:R328/9A* backgrounds, possibly highlighting an influential role for recombination in overcoming the genomic deficiencies shown with these strains. However, based on the lower frequency of recombinants in the *dnaB:muts*, it is likely that unknown suppressors are allowing for growth. To know whether suppressors are present would require significant follow up that we feel may be outside the scope of this manuscript and instead will be addressed in a future manuscript (see comments below). We have experiments planned to test for this possibility, but this determination will likely be several months away. Attempts to make equivalent *recA* knockouts in *dnaB:K180A* strains have not succeeded after four trials, further implying synthetic lethality and survival by suppressors.

Without knowing for certain whether suppressors are allowing for growth in the *dnaB:mut* and *ΔrecA* strains, we are hesitant to include this data in the current manuscript. We actually

wrote this into the revision, but then backed it out because of this uncertainty. On one hand, it is informative that *recA* KOs were more difficult to confirm, but it is difficult to correlate the need for recombination for survival in the *dnaB:mut* strains without validating this specifically by eliminating or confirming the presence of suppressors.

Here is a figure for the validation of the recA KO strains and serial dilution growth assay. Here you can see that adding *ΔrecA* limits growth in this serial dilution assay, however, this is not really all that different from what happens in the *dnaB:WT* strain.

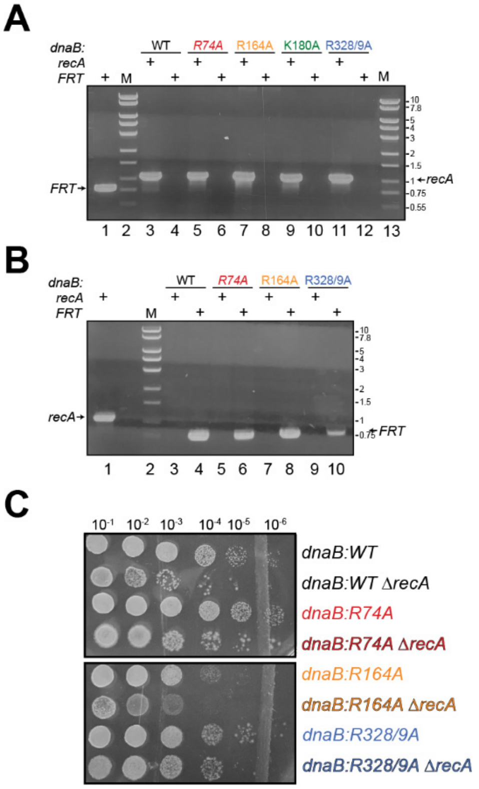

Knockout of *recA* in *dnaB:mut* stains further compromises growth.
recA was deleted in each of the strains: *dnaB:WT*, *dnaB:R74A*, *dnaB:R164A*, or *dnaB:R328/9A* using a FRT-Kan-FRT PCR product with flanking *dnaB* sequences as described in the Materials and Methods. **(A)** Parental strains were screened by PCR for the presence of *recA* and the absence of FRT-Kan as indicated. **(B)** Colonies were selected and screened by PCR for successful knockout of *recA* using primers for *recA* or FRT-Kan. **(C)** Overnight cultures were then serial diluted and grown at 30 ºC. Three biological replicates were performed with a representative plate shown.

The further growth defects seen with knocking out *recA* in these *dnaB:mut* strains and the increased in genomic stress, without inducing the SOS response is still a major unresolved question. Further reverse deletions (*ΔrecA* first followed by *dnaB:mut* edits especially for *dnaB:K180A*) should also be done in addition to testing the RecFOR or PriA/B/C restart pathways through further specific gene deletions. We are planning a follow up manuscript that examines a variety of DNA damage sensitivities and the associated repair pathways with these *dnaB:muts*. Instead of doing significant further work to resolve this in this current manuscript, we respectfully suggest that deciphering the survival mechanism associated

with DNA repair either with RecA, RecFOR, PriA/B/C or something else is instead outside the scope of what we have found here, but we will absolutely follow up on this next.

If the Reviewers still feel strongly that the *recA* KO data is important to include, then it would have to be under the caveat that unknown suppressor mutations may be allowing for growth when *recA* is knocked out.

2. The assessment of the SOS induction is important, but the experimental design used by the authors is complicated. Why is there a need to pre-induce with damage prior to SOS measurement? The cell size heterogeneity and presence of breaks would strongly suggest that authors should be able to measure SOS induction levels without the need for a complicated experimental setup. Can the authors provide measurement of promoter activity in log phase cultures for mutants and wild type from discrete time points without such damage induction (time course is not required for this experiment). This should be corroborated with a Western Blot for RecA/ LexA.
We have repeated the GFP reporter assays for SOS induction in exponentially growing cells with no damage. Interestingly, none of the *dnaB:muts* show any SOS response using our fluorescence reporter assay. It is not until we challenge them with a low dose of MMC does R328/9A appear to have a significant induction over that of the parent strain. We have also included a western blot of LexA that shows stable LexA in all the strains for exponentially growing cells in LB. We have included both of these results in an updated Fig 7.

The absence of a significant SOS response is difficult to reconcile in these *dnaB*:muts strains when we also see significant TUNEL staining, mutagenesis, and increased chromosome content, but we make the case in the Discussion that this may be more consistent with the production of single strand gaps that can be filled in by other more mutagenic Pols (*i.e.* Pol IV) and that these are not large enough for RecA filamentation to reproducibility initiate a full SOS response. Obviously, this will require further work to better characterize the exact repair mechanism, with additional knockouts listed in the response to comment 1 above, but also for Pol II, Pol IV, or Pol IV.

3. The experiment carried out by the authors to determine break formation is relevant and the assay would be robust in the context of DNA damage treatment. However, in context of the dnaB mutants, I wonder whether the lysis technique would impact ssDNA tracts that may be labile to damage. The authors should provide a hydroxyurea-treated control for comparison, where TUNEL labeling should likely not increase more than wildtype (no damage cells).
The lysis procedure was the same for all conditions and strains. The first row in Figure 8 provides an appropriate negative control to account for any protocol specific effects and show very minimal to no BrdU staining, and another row treated with MMC provided a positive control for breaks.

That said, we have also repeated this assay using HU treatment as a third control and see very minimal TUNEL staining by microscopy. This control is now incorporated into Fig 8A. Therefore, it is unlikely that our treatment aberrantly causes breaks.

4. The discussion section would benefit from significant rewriting to compare and contrast, with clarity, the various observations of the mutant phenotypes and the possible mechanisms resulting in the same. The authors should also clearly comment on the extent of ssDNA gaps each mutant might generate and if the same can contribute to the distinct phenotypes observed. Currently, as it is presented, it is unclear as to how and why the perturbations in DnaB toggling between constricted and dilated states results in the myriad phenotypes observed and the model figure does not highlight the impact of the specific mutants in this context.
With the benefit of these additional results, we can now more easily compare and contrast the various *dnaB* mutant phenotypes. Although, there may be other unknown mechanisms for the phenotypic effects seen, we can correlate the degree of constriction in the DnaB hexamer to the most severe genomic deficiencies in the equivalent strains. The Conclusion has been significantly updated to reflect that the hexameric conformation of DnaB is dynamic and that restriction of that conformation presents with a host of genomic instabilities. These most likely consequence is the production of single strand gaps, that are filled in an error prone manner by other polymerases, requiring RecA for efficient growth and survival through various recombination processes that result in increased chromosome content. Again, the mechanism for survival is not fully deciphered, but the characterization of the deleterious effects from single point mutants of *dnaB* are influential.

Minor comment (1): L311 'poles' instead of 'polls'
Corrected

Minor comment (2): Figure S6 is referred to in the manuscript after Fig. S7. Perhaps the order of these figures can be changed.
We have rearranged and updated most of the Supplemental Figures in this revision and have verified that they are correctly referenced.

Reviewer #2:
In this study Behrmann and co-workers describe experiments in E. coli that investigate how DnaB helicase mutants that favour the constricted conformation impact cell growth and genomic stability. The authors show that if a short duplex stretch is located in front of a frayed substrate, the mutants carrying such mutations either have a much-reduced ability to unwind the substrate or cannot unwind the substrate at all, in line with the idea that the constricted conformation for the DnaB helicase is favoured by the mutants. The authors then go on to show that essentially all of the mutants impact the doubling time, and at least some mutants result in the formation of elongated cells. By using direct competition experiments the authors show that mutant cultures are outcompeted by strains carrying the

wild type allele. Run-out experiments with rifampicin show many of the mutants accumulate either more chromosome equivalents or, in addition, complex duplication intermediates. In line with the reduced growth rate and the elongated cells, some of the mutants show an increased mutation frequency, while for the induction of the SOS response more mixed results were found. Finally, the authors directly visualise dsDNA breaks via a bacterial TUNNEL assay and present a molecular model of when the two different states of the DnaB helicase are of particular importance.

Overall, I enjoyed studying the data presented. The authors have produced a large body of work that is well presented and in a logical order, and the questions asked make a lot of sense. However, in its current format the study is not yet scientifically sound, and additional work needs to be done before the work is ready for publication. I hope the comments below will help to improve the work.

Major points

1. In the first paragraph of the Introduction I was slightly lost what the authors actually meant by the term “helicase regulation”. This is then explained as part of the second paragraph, but I wondered whether the integration of what the authors mean, precisely, could be clarified earlier.
Helicase regulation involves the ability to control replication speed to couple DNA synthesis with DNA unwinding and can respond to genomic obstacles, protein interactions, and other coupled events such as priming. We have changed the first sentence in the abstract as well as the last two sentences in the first paragraph of the introduction to make this clearer.

2. Line 103, this section can be improved. For example, an increased SOS response does not necessarily mean increased genomic instability. The authors should take care to be precise about their phrasing, a problem that also applies to a number of other sections (see below).
We have reworded these important final sentences in the introduction to be more precise in our phrasing.

3. Line 130, in line with the comment above, the statement “… in this duplex translocation assay, both K180A and R328/9A mutants are unable to translocate over duplex DNA and therefore maintain a static fully constricted conformation” is not correct, as it is an implication. The assay is indirect, and no direct evidence is presented that the helicase is indeed in the constricted state. I agree with the authors that this is indeed a likely explanation, but the phrasing still needs to be accurate. This also applies to other sections of the manuscript (Line 134 to begin with), including the legend for Figure 1 (Line 682).
OK, added ‘likely’ to account for this implication.

Also changed “Fig. 1: DnaB mutants have impaired ability to translocate over duplex DNA consistent with a more constricted state.”

4. For this particular set of experiments – can the authors rule out that the small unlabelled duplex stretch is not unwound? The entire interpretation is based on this fact. It would be very important to clarify this point, either by clarifying in the text and citing the right source if this was done before, or by showing the relevant data.
This assay was utilized previously by the Berger lab (Ref 14) and although our substrates do not have identical sequences, the general approach is shared and was validated in that reference. In any case, we now provide an additional Supplemental Fig S2 that shows this requested control and conclude that the short (in this case labelled) strand is not unwound by DnaB, but that the fork DNA (in the absence of the short duplex oligo) is unwound.

5. Line 145, why are the authors using the rather unusual temperature of 32°C? Obviously 37°C is the standard temperature used, and 30°C is normally used if temperature-sensitive alleles are used.
These strains are lamda red inducible strains with a CI repressor (CI857) are grown at 30-32C. (We added Ref Thomason 2014 in M&M). We chose 32C for all our assays. Induction of lambda red genes occurs at 42C. This is also why our growth rates are also slower than normal (Fig 2A) to briefly answer your next query.

We added "(to prevent induction of λ red genes)" to clarify the reason

6. There is a problem with the OD measurements, as these are influenced by cell size, and the authors actually demonstrate that, for some mutants, cells are elongated. The same OD for two cultures with different cell sizes means different viable titres. In fact, in Figure 3 the authors show the decline of all mutant cultures relative to cells carrying the wild type allele. Especially for R328/9A how is this possible if the growth rate is not so terribly dissimilar to wild type cells? R164A and K180A are vastly different, but disappear with the same kinetics. This does not make much sense. It could be in part because the cell sizes differ, and for this reason the comparison of the OD readings is not working particularly well, but that would need to be established.
Thank you for pointing this out. As R328/9A has the largest increase in cell size, this may actually be affecting the OD600 readings for growth (Fig 2A-B). We have added a sentence in the Results to explain this caveat.

However, the competition assays in Fig 3 will be able to control for any differences in cell size by counting colonies. In these competition assays, minor differences in growth rates will be amplified during dilution and replating over several days; and so, this assay is really on a different time scale than that in Fig 2A but shows that these strains are compromised for growth overall and easily outcompeted by WT.

Also, the authors should state the more commonly-used doubling time here. The graphs they show highlight steady growth, but the doubling time is very low in comparison to other

experiments, with a doubling time of hours, rather than the usual 20 min for E. coli. This will be, in part, caused by the lower temperature and also by the fact that agitation and therefore aeration is naturally limited in plate readers. Still, in our lab cultures grow in 60 min from 0.2 to 0.8 at 37°C, while they take about 5 h in their set-up. It needs to be highlighted that the growth conditions are rather sub-optimal here.
While it is certainly true, that the conditions for growth are not optimal in the plate reader at 32C, this assay was highly reproducible, internally controlled, and quantitative, allowing us to compare the relative growth rates between strains. Similar results with slower than normal exponential growth rates have been seen by several other labs when measured in plate readers (Ref 48). Slower than optimal growth (similar to our rates) is also seen in these plate reader assays in the following references (though not included in our manuscript).
PMID: 22479509 (Kerner et al PLosOne 2012)
PMID: 33072055 (Bruckbauer et al Front Microbiol 2020)

We added a clarifying sentence in this Results section to account for suboptimal growth conditions. We also changed the time scale to seconds (instead of hours) to be more consistent with the above references and for better comparisons.

7. Do the authors mean "nucleoid", rather than "nucleotide"? Needs correcting throughout.
Thank you for catching this. Corrected three instances.

8. Line 226, the statement here needs either a reference or data. In fact, the same effect would occur if cells have a segregation defect or accumulate replication intermediates that cannot be properly resolved, which would be in line with the observed cell elongation.
We have added several references from work with *dnaA* or *dnaC* mutants that also show intermediate odd numbers of chromosomes. We have also edited this sentence to include the real possibility that they result from unresolvable replication intermediates or defects in segregation in addition to asynchronous initiation.

9. Line 251, if R328/9A has a significantly reduced loading efficiency how come it is growing so relatively fast?
These correlations are not always direct. We can measure a reduced *in vitro* loading efficiency for DnaB(R328/9A), but this would be more difficult to measure *in vivo* (although this is something we are considering for the future). And although DnaB loading at oriC should ideally correlate with slower growth, this mutant has the fastest in vitro DNA unwinding rate of all the mutants (Carney et al. 2017 JBC). So, the affect may be tempered by these facets as well as many other unknowns.

10. Line 264, all experiments so far were done in rich medium as far as I can see. Now the authors suddenly switch to M9. Why the change? Rif rates can be established perfectly fine in rich medium as well.

Rifampicin mutagenesis assays are commonly performed in M9 to induce some level of stress. However, in light of this comment, we have repeated these assays in rich media (LB). Fig 6C has been updated to include Rif frequencies in rich media, and the previous plot performed in M9 has been removed. We agree this fits better with the rest of the story throughout. Moreover, we added whole genome sequencing data for all of the strains that indicate they are mutator strains in rich media which aligns with this comment. See Response to Reviewer 3 below.

11. Line 266, it is extremely important that the authors use precise language here. What they have established is *not* a mutation rate. Instead, they have measured frequencies, for which they calculated the arithmetic mean (see below). I believe in the M&M section this was correctly described; it cannot be called a rate here. Also, for a frequency the authors cannot, and should not, calculate the arithmetic mean. Mutation rates are established by a rather elaborate fluctuation analysis, which requires the growth of multiple parallel cultures (11 or more). I am not saying that the authors need to establish rates here, but when doing a fluctuation analysis, it becomes obvious that in some cultures the first event is, by chance, happening early, leading to a culture with very high numbers of Rif-resistant cells. In a fluctuation analysis these cultures will be deliberately discarded. However, by calculating the mean the authors include what can be a vast variability which is *not* representative for the actual mutation rate in their calculations. It would be much better to show individual data points here, rather than an average.
Absolutely correct, and this was an oversight on our part to not be internally consistent. We have corrected all of this language to only include 'measured frequencies'. The frequencies or "Mutants pre 10^6 cfu" were plotted in Fig 6C as individual data points. Although we did see the most variability in dnaB:R328/9A, the rest of the mutant frequencies cluster. Therefore, we also included a shaded bar that represents the mean for easy comparison.

12. Line 289, why was MMC included here? And why are experiments without MMC not shown?
Please see response to Reviewer 1, comment 2. This has been updated.

13. Line 304, the authors use the term "Log phase cultures" here and elsewhere. While very common, it is not good practice to use the term "logarithmic growth". What is that supposed to be? While understandable for most readers, the term "exponential growth" is a much better choice and should be used throughout the manuscript.
Thank you for this explanation. Log phase has been changed to "exponential growth" or " exponentially growing" throughout.

14. Line 484, the composition of "LB" needs to be defined here, as there are at least three different recipes which differ (mostly) in salt content.
Added

15. Line 521, the procedure here needs to be clarified. How exactly were the cultures treated? Were cells passaged to refresh growth? And if so when were the dilution steps done?
The procedure was updated in the Materials and Methods to be more specific with regards to the specific dilutions and growth conditions.

Minor points
Line 40, an "all" is missing before "Domains", and the latter should be lowercase.
fixed
Line 165, "Large" should be lowercase.
fixed
Line 311, I believe the authors mean "poles", rather than "polls".
fixed

Line 348, I believe this statement needs a reference.
This sentence is ours and does not necessary rely on any particular reference. "However, while one function of helicase regulation is clearly to limit ssDNA production, a lack of increased SOS response for most of the mutants indicates that the function of helicase regulation goes beyond limiting unwinding and impacts other aspects of replisome activities."

Line 359, what is "G. ste" supposed to mean?
Geobacillus stearothermophilus
Fixed

Line 735, the grammar is not quite right here.
fixed

Line 736, "preequilibrated" needs a hyphen
fixed

Reviewer #3:
In this manuscript the authors follow on from their previous work to try to further characterize a set of surface residue mutants of DnaB. In previous work it was suggested that these mutants affect the interaction of the excluded strand of ssDNA with the surface of DnaB leading to a loss of control of helicase activity.
In this work the authors suggest that these mutants alter the ability of the N-terminal domain of DnaB to undergo a transition from a constricted to dilated state, based on the inability of the mutant DnaBs to pass over a section of dsDNA.
The work then looks at in vivo effects of introducing these mutants into E. coli cells by a number of assays. The phenotypes are quite complex and not clear-cut, making interpretation tricky. However, it is clear that the mutations are deleterious, lead to

chromosomal abnormalities, increased mutation rates, increased DNA content in cells and chromosome segregation problems.

One suggestion for an improvement would be to have reference to Figure S8 much earlier, so that it is more obvious where the mutants that are used in the study map to. I found myself wanting a figure like this from the introduction but only discovered it was present by the discussion.
Agreed. This is now Figure S1. And all the other Supplemental Figures have been adjusted.

Specific comments:

Heading: “Introuction” should be “Introduction”
Fixed

Introduction, line 55: is “proximal” the right word? In this case do you mean central or vital to the process, rather than adjacent to?
Proximal means ‘central’ or ‘center of the body’. This is what we are trying to convey.

Line 58 should be “unwinding by…” rather than “unwinding of”
Fixed

Line 66 “helicase mechanism dysregulation”- could be just “helicase dysregulation”
Agreed

Line 77-79 “This regulation may be important *in vivo* to limit separation of DnaB from the replisome, which may occur during Okazaki fragment priming or during helicase-polymerase decoupling [22, 23].” Are these the correct references here? The Mangiameli et al paper discusses helicase transcription conflicts but I don’t recall any mention of uncoupling of helicase and polymerases or primase? Similarly in the Nature paper I don’t recall these points being discussed?
Thanks for catching this. We have removed the Mangiameli reference 22 that was improperly placed. Instead, we have added three new references (to go with the original Fijalkowska reference 23) now 22-25 that highlight frequent Pol III exchange/switching that occurs routinely, a stable DnaB, and fast highly processive replication when Pol III is coupled with DnaB.

Making site-specific mutants: one problem in making mutants in an essential gene is the large selection pressure for suppressor mutants in alleles which are deleterious. It would be good to know how easily each SEW mutant could be transduced into a wt strain (or alternatively to see the whole genome sequence of the mutants compared to their parental strain to detect suppressors).

One of the main points of this work is to show that CRISPR-Cas9 editing is faster and easier than transduction. But we appreciate the comment, and we had also thought of this. But we decided to go with the strategies below instead.

Of course, the CRISPR approach used does not generate an associated selectable marker, so transduction would not be easy.
True, which is why the transduction approach was not the easiest and we went with CRISPR editing instead.

One possible indication of whether each mutation is deleterious is the relative efficiency that changes were detected in the dnaB gene following the CRISPR plasmid induction. Was each mutant detected with the same efficiency, or were some harder to make than others?
We do have numbers on this, and we have now included them in S3 Fig. legend and in the text C). Essentially all of the mutations were easy to make. We had several positive colonies in each experiment with very low background. Of course, these were initially screened by restriction digest, but we also sequenced several isolates and got the same mutations in *dnaB*. And our newly added WGS further confirmed these *dnaB* mutations.

One concern is that, in vitro, constricted mutants did not show lagging strand synthesis (Monachino et al. 2020). A downregulation of lagging strand synthesis in vivo could account for the observed growth defect. However, if serious, then it could also lead to compensatory mutations in other genes to overcome this pressure. This possibility (compensatory secondary mutations) should be discussed, or evidence provided for why this is not a concern.
This is an interesting point that we had also considered. It may be that gaps are occurring preferentially on the lagging strand, but that is a bit outside the scope of this initial manuscript, as we discuss below.

However, we have (in collaboration with new authors Visser and Bates) sequenced our initial *dnaB:mut* strains. Although there are not obvious suppressors that would promote replication, Okazaki fragment maturation, or recombination, we did discover that all strains appear to be mutator strains with several mutations across the genome (new S1 Table, Fig 6A-B). This is now discussed in greater detail both in the Results and Conclusions sections.

Growth rate experiments: what do the growth rate calculations mean, because visually they do not make much sense when you look at the exponential phase of the growth cycle: for example the wt goes from an OD of 0.2 to 0.4 in about an hour (i.e growth rate of 1 per hour?). It then goes from 0.4 to 0.8 in just over 2 hours. How does the growth rate come out to be 0.18? It does not seem to match with the actual doubling time of the cultures shown at exponential growth, but is designed to account for an increased lag phase as well? Perhaps a clearer explanation of the single growth rate parameter quoted would make it easier for readers to interpret the data.

Understood. This is also related to Reviewer 2 Comment 6. Please see our response there. We have recalculated these rates to be min^{-1}. We have also provided several references that also show slower overall growth rates within the confines of 96 well plate readers and are similar to what we measure.

The micrographs in Fig. 2 (even with the zoomed inserts) are too small and low quality to properly make out necessary details. Do the filaments have segregated nucleioids or not? This would be very interesting to know.
We have updated Fig 2 to have better zoomed in images. Of course, we were trying to give a broader perspective, but we may have missed the mark initially. These new images more clearly show the elongation phenotype with several of the strains. The original figures are now included within Supplemental S4 Fig. We do not have the resolution of staining to conclude whether there are segregated nucleoides, but we agree this would be interesting and something to investigate in the future.

Line 196 –"least" rather than "lease"
Fixed

Line 227: "route causes"- I think you mean root causes.
Fixed

Ori:ter ratios. While this is generally supportive of a defect in these cells, it is likely that a delay in cell division due to some filamentation can cause these changes. To my mind, an ori:ter ratio of 3 in cells is an average of recently divided cells which have a ratio of 2:1 and pre-division cells which have an average of 4:1. If division is delayed then there are more 4:1 cells, possibly even 8:1 and fewer 2:1. The altered ratios in the mutants may just reflect delayed division?
This may also possible. We have updated the text to include this possibility. However, the increase in *ori:ter* ratios really represents a delay in termination of replication or segregation and not initiation. Furthermore, we are not aware of any examples where cell division delays (e.g. FtsZ(Ts) or cephalexin), affect ori-ter ratios. Rather the increase in ori:ter seen with these dnaB:muts likely represent some delays in elongation, that impact termination and segregation while allowing more Ori's to be active.

What happens with exponentially growing cells by FACS? In wt cells you usually see a signal something like this: a peak at 2 chromosome copy number then an exponential decrease towards 4 copies per cell. If the mutants have problems with replisome stalling then there might not be the exponential decrease seen in wt. It would be interesting to see if introducing a mutation that destabilized RNA polymerase (eg rpoB*35) increased the fitness of these mutants, or if the fitness loss is not related directly to replisome collisions/collapse.
Of course, we have done FACS analysis of chromosome content using the rifampicin/cephalexin run out assay (Fig 4A), which shows both distinct cell populations with

greater than 2 or 4 chromosome as well as more diffuse peaks throughout indicating extreme cell chromosome complexities.

We have performed FACS on exponentially growing cells however, we were unable to obtain any distinct resolution of peaks as you suggest. There are some indications of bumps in the histograms that could be consistent with chromosome peaks, but the diversity here is just too great. Interestingly, *dnab:K180A* has a broader and larger histrogram distribution than the *dnaB:WT* or even the rest of the *dnaB:muts*. However, we feel as though the data presented in Fig 4A more clearly makes the case for the increase in chromosome complexity seen in these strains when we can limit further initiation events.

SOS induction and BrdU incorporation: it would also be interesting to see the relative viability/fitness of a recA mutant in the dnaB mutant backgrounds.
Agreed, and with your comment along with Reviewer 1 comment 1, we have created *recA* knockouts in our *dnaB:mut* strains. Please see our response there. But adding *recA* deletion was only possible in 3 out of 4 of the *dnaB:muts*, and we cannot rule out, at this point, that survival depends on unknown suppressor mutations in the delta-recA strains. However, the growth rates were slower indicating RecA has a role, but survival may (or may not) be completely dependent on recombination, and unfortunately we are not able to say with certainty yet.

Line 336-338 and following sentences: “DnaG, which favors the dilated state of DnaB, has been shown to limit replisome progression and generate pausing events [47], consistent with a model where the helicase constricts”. It has been shown that in the presence of the clamp loader complex there is no pausing required for DnaG action and that there is likely a spiral, non-planar N terminus state (Monachino et al., 2020)
While it is true that Monachino et. al. show that a crosslinked DnaGC-DnaB construct remains an active replication complex, these bulk assays were performed with a destabilized replisome in the absence of the X and Ψ components of the CLC. Additionally, these assays were designed to allow for leading strand synthesis only; it is possible that while simply binding to DnaGC-term does not limit activity, forming a complex with active, priming DnaG monomers does effects helicase progression as shown for T7 (Jong-Bong Lee et. al. 2006).

These same authors showed previously that helicase-primase interaction reduces replisome processivity (Tanner et. al. 2008). It is also understood that depending on reaction conditions, DnaG can stimulate helicase activity, and this net stimulation would conceal any brief pausing events when investigated in a bulk assay. We recognize that this topic is still under investigation, and there are complex results and opposing views. The comment is appreciated, and the text has been updated to be more specific with language and contain additional references, but the absolute effects of DnaG on replisome progression, especially when altering the DnaB conformation, are still under investigation.

Line 379-80: “ori and/or ter regions of the chromosome, which migrate to the ends of the cell during chromosome segregation [52-54]” Is this is true? In wt E. coli the ori region starts at midcell and then migrates to the ¼ and ¾ positions. The terminus region can be polar then migrates to midcell for duplication and remains there prior to segregation (for example see Wang, Possoz and Sherratt, 2005).
This is basically the same point we were trying to convey. Interestingly, the TUNEL foci we see in *dnaB:R74A* are consistently at the “terminal poles” which would be consistent with issues at the ori (Fig 8A, 4th panel). In cells captured just after cell division, there is no staining at the fision points. Of course, this is just an observation, and we are following up on this for a future manuscript.

Line 419: would “harmony” be better than “symphony” here?
Yes. "harmony” this has been changed.

Line 570: 0.5 µL each of 100 µM forward and reverse primers. Is this correct- most often protocols would use 0.5 µL of 10 µM primer.
Our mistake, and you are correct. 0.5 µL each of 10 µM primers

Figure S1: part A: the lower-right red arrowhead, showing the BsaI cleavage site is one base out from where it should be. It should cleave after AAAA (4bp overhang).
This is now S3 Fig. This is a great catch. This arrow did get moved somehow in the figure that we uploaded. This has now been corrected.

Fig S3. The circled small populations in the last 3 mutants- it is not convincing that these are real cells rather than some kind of background. In microscopy, were any small or mini cells (no DNA content) seen that could account for these populations (using light microscopy rather than the DAPI stain)?
We looked for anuclear cells by microscopy and did not see any obvious candidates. Therefore, we have removed the circles on S4 Fig. and references to this minor population. Instead, we have focused on the populations described in Fig. 4 to show increased chromosome content.
